# Complement Factor B Deficiency Is Dispensable for Female Fertility but Affects Microbiome Diversity and Complement Activity

**DOI:** 10.3390/ijms26031393

**Published:** 2025-02-06

**Authors:** Manato Sunamoto, Kazunori Morohoshi, Ban Sato, Ryo Mihashi, Masafumi Inui, Mitsutoshi Yamada, Kenji Miyado, Natsuko Kawano

**Affiliations:** 1 Laboratory of Regulatory Biology, Department of Life Sciences, School of Agriculture, Meiji University, 1-1-1 Higashimita, Kawasaki 214-8571, Japan; cf240412@meiji.ac.jp (M.S.); kmorohoshi@ndmc.ac.jp (K.M.); bansato@meiji.ac.jp (B.S.);; 2 Division of Biomedical Engineering, National Defense Medical College Research Institute, 3-2 Namiki, Tokorozawa 359-8513, Japan; 3 Laboratory of Animal Regeneration Systemology, Department of Life Sciences, School of Agriculture, Meiji University, 1-1-1 Higashimita, Kawasaki 214-8571, Japan; inui_m@meiji.ac.jp; 4 Department of Obstetrics and Gynecology, Keio University School of Medicine, 35 Shinanomachi, Shinjuku, Tokyo 160-8582, Japan; mitsutoshi.yamada@gmail.com; 5 Department of Reproductive Biology, National Research Institute for Child Health and Development, 2-10-1 Okura, Setagaya, Tokyo 157-8535, Japan; miyado-k@ncchd.go.jp

**Keywords:** complement factor B, alternative pathway, mouse fertility, gut microbiome, vaginal microbiome

## Abstract

Complement factor B (CFB) is a crucial component for the activation of the alternative pathway due to the formation of the C3 convertase with C3b, which further produces C3b to enhance the overall complement activity. Although *Cfb* is expressed not only in the immune tissues, but also in the reproductive tract, the physiological role of the alternative complement pathway in reproduction remains unclear. In this study, we addressed this issue by producing *Cfb*-knockout (KO) mice and analyzing their phenotypes. Sperm function, number of ovulated oocytes, and litter size were normal in KO mice. In contrast, the diversity of microbiomes in the gut and vaginal tract significantly increased in KO mice. Some serine protease activity in the serum from KO mice was lower than that of wild-type mice. Since the serum from KO mice showed significantly lower activity of the alternative complement pathway, CFB was found to be essential for this pathway. Our results indicate that although the alternative pathway is dispensable for normal fertility and development, it maintains the gut and vaginal microbiomes by suppressing their diversity and activating the alternative complement pathway.

## 1. Introduction

The complement system is a proteolytic cascade of the innate immunity, that plays an important role in the recognition and elimination of pathogens throughout the animal kingdom [1]. This system can be activated through three pathways, depending on the pathogen recognition pattern: (1) classical, (2) lectin, and (3) alternative. All three pathways require C3 convertase, belonging to the family of serine proteases, to generate active C3b from native C3.

The classical pathway is activated using antigen–antibody complexes, whereas the lectin pathway is activated by the recognition of specific glycan structures. Previous studies have shown that *C1q*-KO mice, which lack the classical complement activity, exhibit delayed recovery after spinal cord injury [2]. Similarly, mannose-binding, lectin-deficient mice, which lack the functional lectin pathway activity, are more susceptible to infection with *Staphylococcus aureus* [3].

In contrast, the alternative pathway is triggered by spontaneous hydrolysis of C3 to C3(H_2_O), in the absence of specific activating factors. The generation of the “C3b-like” C3(H_2_O) forms the initial C3 convertase with complement factor B (CFB). This reaction occurs constantly at low levels in the blood and is also known as the tick-over theory [4,5]. This system has been detected in vitro and in vivo, and has accelerated even when the blood is exposed to nonspecific activators, such as oxygen bubbles [6,7].

The alternative pathway shifts the C3b production, from small amounts during homeostasis, to large amounts during emergencies, suggesting that it plays an important role in the early stages of infection. Thus, the three complement pathways act as part of the innate immune system in defense against infection from pathogens, with the alternative pathway specifically involved in the surveillance of potentially invading pathogens, and contributing to a rapid amplification loop during bacterial infection.

A close relationship exists between the immune system and the female reproduction. The defensive immune responses in the female reproductive tract eliminate the invading pathogens and are tolerant to symbiotic bacteria, sperm, and fetuses [8]. Symbiotic bacteria exist in the vagina and act as biological defenses. The uterine microbiome is also involved in fertility and uterine diseases in humans [9]. Recently, it has been suggested that gut microbial homeostasis is essential for reproductive health [10]. A balance between the immune response and the tolerance is necessary for both the selection of symbiotic bacteria and successful pregnancies. In particular, complement components are critical for pathogen elimination, while sparing commensal organisms in the gut microbiota [11]. Additionally, they play a crucial role in the implantation and placental development at the maternal-fetal interface [12,13,14,15]. Although the activity of the alternative pathway is increased in the mouse placenta from spontaneous abortions [16], the precise mechanism between the alternative pathway and pregnancy is unclear.

CFB, an 85-kDa serine protease, is essential for activating the alternative pathway. CFB allows the deposition of C3b on the pathogen surface, and the bound CFB is activated by complement factor D, generating CFBa and CFBb. The Bb fragment, which has a serine protease domain, remains bound to C3b, and forms C3bBb, a C3 convertase in the alternative pathway. This enzyme amplifies C3b production and enhances the overall effects of the complement system. However, in a previous study, a CFB deficiency caused no dramatic abnormalities, despite the low activity of the alternative pathway [17]. There are still many unknown factors regarding the physiological role of the alternative complement pathway in reproduction, owing to the lack of reports on the phenotype of *Cfb*-knockout (KO) mice. In this study, we generated KO mice with low alternative pathway activity, and examined their gut and vaginal microbiota, as well as their reproductive functions.

## 2. Results

### 2.1. Cfb Expression in Reproductive Tissues

**Figure 1:**
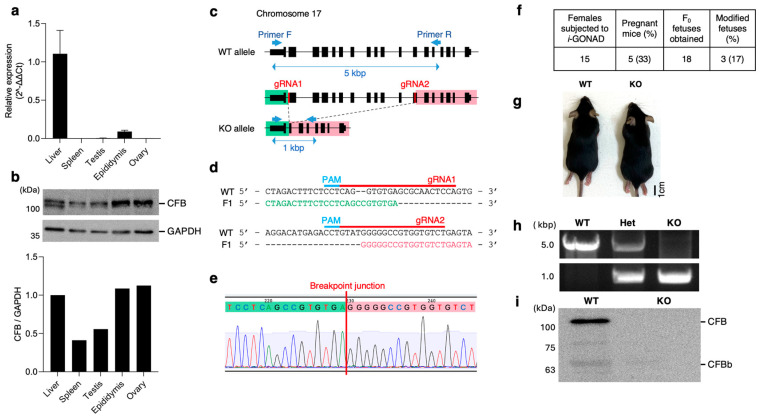
*Complement factor b (Cfb)* expression analysis and generation of *Cfb*-KO mice. (**a**) *Cfb* mRNA expression in several tissues was analyzed by qPCR. *n* = 3. Data are expressed as means ± SE. (**b**) Immunoblotting of several tissue lysates with anti-CFB Ab and anti-GAPDH. GAPDH is an endogenous control. The densitometry analyses of band intensity normalized with GAPDH. (**c**) Schematic illustration of the wild-type (WT) and *Cfb*-KO (KO) allele of the *Cfb* gene. Black boxes mean exons. We designed gRNAs at exon 2 and exon 12, and KO allele deleted a 4 kbp long sequence in chromosome 17. (**d**) Sequences of PCR products with using Primer F/R in an WT and F1 mouse. gRNA sequences were found in WT allele, but not in F1 allele. Green characters indicated intron sequences between exon 1 and 2, and pink characters means exon 12 sequence. (**e**) Genomic DNA sequence at breakpoint junction in KO mice. Green and pink shading characters indicate the intron between exon 1 and 2 and the exon 12, respectively. (**f**) Efficiencies of *i*-GONAD treatment. (**g**) Representative image of WT and KO mice. (**h**) Genotyping of the F2 by using primer F and R. (**i**) Immunoblotting the serum from KO mice with anti-CFB Ab.

*Cfb* mRNA expression in immune and reproductive tissues from wild-type (WT) mice was examined using qPCR. CFB was expressed mainly in the liver, but slight expression levels were also observed in the epididymis, testes, and ovaries (Figure 1a). Immunoblotting of tissue lysates was also performed (Figure 1b). CFB proteins were detected in all lysates, with particularly intense signals in the epididymis and ovary; however, the amounts were not consistent with the mRNA levels. These results indicate that the CFB protein is present in all tissues examined, regardless of mRNA expression, and especially in the reproductive organs.

### 2.2. Generation of KO Mice

We generated mice lacking *Cfb* using improved genome editing via oviductal nucleic acid delivery (*i*-GONAD) methods [18]. The mouse *Cfb* gene is located on chromosome 17 and comprises of 18 exons. We designed two gRNAs, targeting exons 2 and 12, and performed *i*-GONAD in C57BL/6N mice (Figure 1c). The sequencing of genomic DNA, isolated from one of the F1 offspring, revealed 3833 bp deletion in the mutated allele (Figure 1d,e). Five of the fifteen females subjected to *i*-GONAD delivered pups (Figure 1f). The genotyping of the F0 offspring demonstrated that three of the eighteen F0 pups (17%) had a mutated allele. The homozygous (*Cfb*-deficient; KO) mice were healthy and grew normally (Figure 1g). These mice were obtained by crossing F1 and identified by a PCR analysis of their genomic DNA (Figure 1h). The CFB protein completely disappeared in the serum of KO mice (Figure 1i).

### 2.3. Fertility of KO Mice

**Figure 2:**
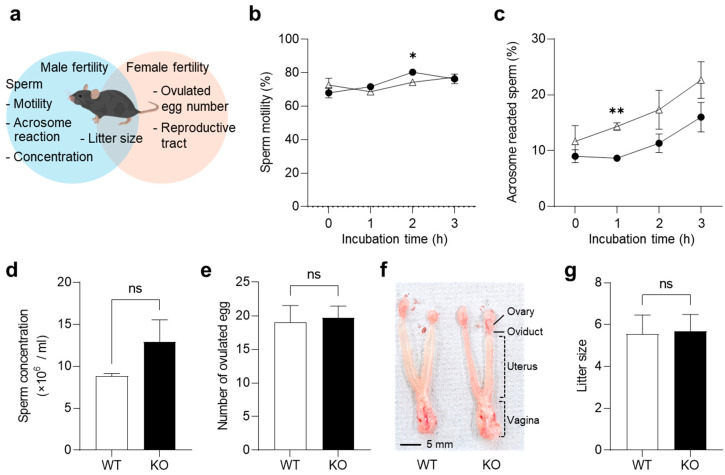
Fertility of KO mice. (**a**) A method for measuring mouse fertility. For the analysis of male fertility, cauda epididymal sperm were collected from male KO mice. After the incubation in TYH medium, sperm were subjected to sperm motility and concentration analysis. For female fertility, Cumulus-oocyte complexes were isolated from super-ovulated KO mice. The number of pups produced after mating was counted to determine the reproductive potential of both males and females. (**b**) Sperm motility during 3 h of incubation between WT mice (triangles) and KO mice (solid circles): * *p* < 0.05. *n* = 3. Data are expressed as means ± SE. (**c**) Rates of acrosome reacted sperm during 3 h of incubation between WT mice (triangles) and KO mice (solid circles): ** *p* < 0.01. *n* = 3. Data are expressed as means ± SE. (**d**) Epididymal sperm concentration: ns, not significant, *n* = 3. Data are expressed as means ± SE. (**e**) The number of ovulated eggs: ns, not significant, *n* = 3. Data are expressed as means ± SE. (**f**) Representative image showing an entire reproductive tract of estrous female. (**g**) Fecundity of KO mice: ns, not significant, *n* = 3. Data are expressed as means ± SE.

Since CFB was abundantly expressed in the reproductive organs (Figure 1), we examined the abnormalities in cauda epididymal sperm and the ovulated eggs from KO mice (Figure 2a).

To examine the sperm maturation, epididymal sperm were collected from the cauda epididymis, and their motility was measured after in vitro incubation (Figure 2b). Sperm from KO mice exhibited a significantly higher motility than those from WT mice after 2 h of incubation (*p* = 0.017), but no significant differences were observed after 0, 1, and 3 h of incubation. Next, the percentage of the spontaneous acrosome reaction was measured (Figure 2c). Sperm from KO mice showed lower rates of acrosome reaction than those from WT mice at each time point, especially after 1 h of incubation (*p* = 0.0022). The concentration of epididymal sperm was not significantly different between the WT and KO mice (*p* = 0.058; Figure 2d). These results suggest that a CFB deficiency does not affect spermatogenesis or sperm maturation.

We examined the effects of CFB on the female ovulation. Following superovulation, eggs were isolated from the oviducts of WT and KO female mice. There were no significant differences in the number of ovulated eggs from WT mice (19.0 ± 2.1) and KO mice (19.7 ± 1.4; *p* = 0.84; Figure 2e). The reproductive tracts (vagina, uterus, and oviduct) and ovaries were isolated from estrous females, but no overt abnormalities were found in their appearance (Figure 2f). Finally, we investigated the fertility of the KO mice. The average litter size did not differ between the WT and KO mice (Figure 2g). These results suggest that CFB is involved in sperm function, but is not essential for fertility.

### 2.4. Gut and Vaginal Microbiome of KO Mice

**Figure 3:**
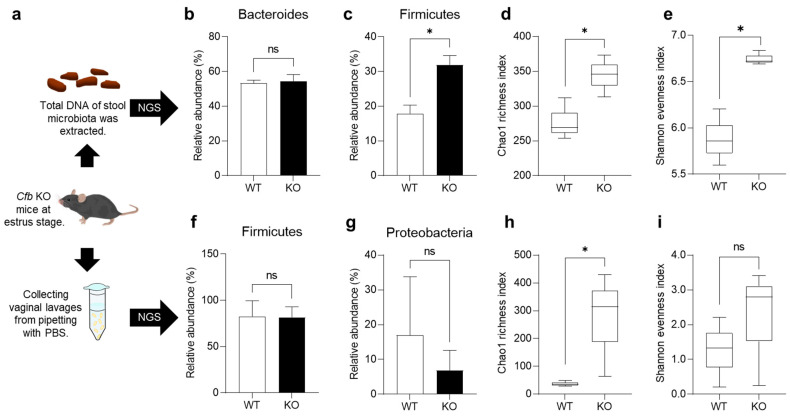
Characterization of gut and vaginal microbiota. (**a**) Stool samples and vaginal lavages were collected from identical mice at the estrous stage. (**b**) Relative abundance of *Bacteroides* in the gut microbiota: ns, not significant, *n* = 3. Data are expressed as means ± SE. (**c**) Relative abundance of *Firmicutes* in the gut microbiota: * *p* < 0.05, *n* = 3. Data are expressed as means ± SE. (**d**) Total alpha diversity (Chao 1) of the gut microbiota: * *p* < 0.05, *n* = 3. Boxes represent the interquartile range, lines indicate medians, and whiskers indicate the range. (**e**) Total alpha diversity (Shannon) of the gut microbiota: * *p* < 0.05, *n* = 3. Boxes represent the interquartile range, lines indicate medians, and whiskers indicate the range. (**f**) Relative abundance of *Firmicutes* in the vaginal microbiota: ns, not significant, *n* = 3. Data are expressed as means ± SE. (**g**) Relative abundance of *Proteobacteria* in the vaginal microbiota: ns, not significant, *n* = 3. Data are expressed as means ± SE. (**h**) Total alpha diversity (Chao 1) of the vaginal microbiota: * *p* < 0.05, *n* = 3. Boxes represent the interquartile range, lines indicate medians, and whiskers indicate the range. (**i**) Total alpha diversity (Shannon) of the vaginal microbiota: ns, not significant, *n* = 3. Boxes represent the interquartile range, lines indicate medians, and whiskers indicate the range.

There is a close relationship between complement activity and the symbiotic microbiome [19]. To investigate the effects of a CFB deficiency on the microbiome, we analyzed the gut and vaginal microbiomes of KO mice using 16S rRNA gene amplicon sequencing (Figure 3a).

To analyze the gut microbiome, we generated a total of 256.2 thousand high-quality reads with a mean (standard deviation, SD) of 42.7 (5.70) thousand reads for each sample (Supplementary Table S1). The most abundant gut microbiota in each subject at the phylum level (top nine) are shown in Supplementary Figure S1a. The dominant phyla in the WT and KO mouse groups were *Bacteroidetes* and *Firmicutes*. The majority of *Bacteroidetes* in KO mice (54.5%) was comparable to that in WT mice (53.2%; *p* = 0.95; Figure 3b). In contrast, the Firmicutes population significantly increased in KO mice (WT, 17.9%; KO, 31.9%; *p* = 0.019; Figure 3c). The total alpha diversity of the gut microbiome tended to be higher in KO mice than in WT mice, as indicated by Chao1 (*p* = 0.049; Figure 3d) and Shannon (*p* = 0.049; Figure 3e). The total beta diversity of the gut microbiome was not different between WT mice and KO mice (*p* = 0.10; Supplementary Figure S2a,b).

To analyze the vaginal microbiome, we generated 250.1 thousand high-quality reads with a mean (SD) of 41.7 (6.84) thousand reads for each sample (Supplementary Table S2). The most abundant vaginal microbiota in each subject at the phylum level (top eight) are shown in Supplementary Figure S1b. The dominant phylum in both WT and KO mouse groups was *Firmicutes*. There was no significant difference in the major population of *Firmicutes* (WT, 82.7%; KO, 81.7%; *p* = 0.96; Figure 3f) and *Proteobacteria* (WT, 17.0%; KO, 6.9%; *p* = 0.59; Figure 3g) between the two groups. In addition, the total alpha diversity of the vaginal microbiome was higher in KO mice than in WT mice, as indicated by Chao1 (*p* = 0.049; Figure 3h). In contrast, Shannon exhibited no significant difference between that of WT mice and KO mice (*p* = 0.27; Figure 3i). The total beta diversity of the vaginal microbiome was not different between WT mice and KO mice (*p* = 0.10; Supplementary Figure S2c,d). These results indicate that CFB deficiency enhances the microbiome diversity in both the gut and vagina of mice.

### 2.5. Proteolytic Activity in the Serum from KO Mice

**Figure 4:**
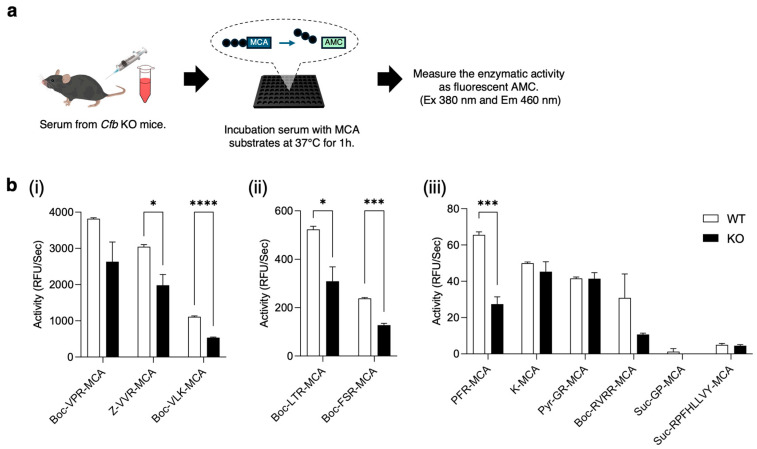
Enzyme assay of serum from KO mice. (**a**) Experimental flow. Serum was collected from wild-type and KO mice and then incubated with various MCA substrates at 37 °C for 1 h. Released fluorescence was measured every 10 min for 1 h. (**b**) Substrates with strong enzyme activity (**i**), with moderate enzyme activity (**ii**), with weak enzyme activity (**iii**). * *p* < 0.05, *** *p* < 0.001, **** *p* < 0.0001. *n* = 3. Data are expressed as means ± SE.

As CFB is a serine protease, its deficiency may affect the activities of other proteases. To estimate the proteolytic activity in the serum, we used various fluorogenic substrates (Figure 4a; Supplemental Table S3). Serum from WT mice showed strong activity against Boc-VPR-MCA, Z-VVR-MCA, and Boc-VLK-MCA, whereas the serum from KO mice showed a significantly lower activity against Z-VVR-MCA (*p* = 0.025) and Boc-VLK-MCA (*p* = 0.000041; Figure 4b(i)). Among the substrates with a moderate activity, the serum from KO mice exhibited significantly lower activity against Boc-LTR-MCA (*p =* 0.024) and Boc-FSR-MCA (*p* = 0.00016; Figure 4b(ii)). Among substrates with weak activity, only in PFR-MCA, the enzyme activity was significantly different between the serum from WT and KO mice (*p* = 0.00091; Figure 4b(iii)). These results indicate that CFB deficiency affects several proteolytic activities in the serum.

### 2.6. Hemolytic Assay of the Serum from KO Mice

**Figure 5:**
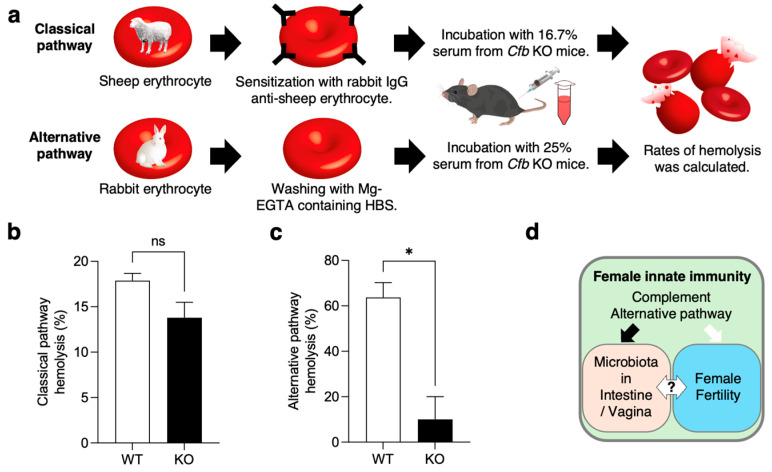
Hemolysis assay of serum from KO mice. (**a**) Experimental flow. For measuring hemolytic activity of complement classical pathway, sheep erythrocyte was sensitized with rabbit IgG and then incubated with the serum from WT and KO mice. To measure that of complement alternative pathway, rabbit erythrocyte was incubated with the serum in the presence of EGTA. After incubation, the rates of hemolysis were calculated. (**b**) Classical pathway hemolytic activity: ns, not significant, *n* = 3. Data are expressed as means ± SE. (**c**) Alternative pathway hemolytic activity: * *p* < 0.05, *n* = 3. Data are expressed as means ± SE. (**d**) Schematic model of the relationship between innate immunity and the microbiota in female mice. The relationship between microbiota diversity and female fertility remains unclear.

To confirm the complement activity of serum from KO mice, we performed hemolytic assay (Figure 5a). Classical pathway activity was assessed using sensitized sheep erythrocytes. Although the serum from KO mice (13.8 ± 1.7%) showed lower activity than that from WT mice (17.9 ± 0.8%), there were no significant differences in classical pathway hemolysis between the two strains of mice (*p* = 0.095; Figure 5b).

Alternative pathway activity was assessed in rabbit erythrocytes. The serum from KO mice had a significantly lower activity of the alternative pathway (10.1 ± 8.3%), compared to that from WT mice (63.9 ± 5.3%; *p* = 0.016; Figure 5c). These results indicate that alternative pathway activity is significantly lower in serum lacking CFB.

## 3. Discussion

In the present study, we generated KO mice and analyzed their reproductive and microbial phenotypes. Our results demonstrated that *Cfb* is expressed not only in the immune organs, but also in the reproductive organs. However, we found no significant changes in sperm and oocyte function, and observed normal litter production in KO mice (Figure 2). These results suggest that the CFB is dispensable for normal fertilization, including sperm-egg adhesion, fusion, and normal development. In contrast, significant differences were observed in the microbiomes present in the gut and vaginal tracts (Figure 3). In KO mice, the microbiome diversity was significantly increased in both organs. The vaginal microbiome is known to be influenced by the estrous cycle, and its composition varies greatly among individuals [20,21]. In this study, by adjusting the estrous cycle, using vaginal smears, we were able to compare between the mice and detect the differences in the vaginal microbiome. These suggest that the alternative pathway regulates microbiome diversity throughout the body (Figure 5d).

In the gut microbiota, the abundance of *Bacteroidetes* (the major population) remained unchanged, but the abundance of Firmicutes (the secondary population) increased in KO mice. It was already known that the population of *Bacteroidetes* increased, whereas that of *Firmicutes* decreased, in *C3*-deficient mice [22]. Complement classical and/or lectin pathways may control the abundance of *Bacteroidetes* because a CFB deficiency had no effect on them. In contrast, the alternative pathway is thought to regulate the population of *Firmicutes*, as increased levels were only observed in the KO mice. This indicates that each complement pathway regulates the balance of the gut microbiota differently.

Further analysis of the vaginal microbiome of the KO mice revealed a predominance of *Firmicutes*. In general, the human vaginal microbiota, which is composed of *Lactobacillus* spp. and lactic acid-mediated vaginal acidification, is important for protection against sexually transmitted pathogens and opportunistic infection [23,24]. Similarly, the mouse vaginal microbiota is known to be dominated by *Firmicutes* phyla, despite its near-neutral pH [25,26]. Although the abundance of *Firmicutes* did not change between the WT and KO mice, the vaginal microbiome diversity increased in KO mice. A high vaginal bacterial diversity in women at a high risk of a spontaneous preterm birth leads to increased MBL, IgG, C3b, and C5 concentrations [27]. Furthermore, the classical and alternative pathways are activated in the vagina during pregnancy and labor, and alternative pathway activation is well-controlled during delivery [28]. Further analysis also showed that the activation levels of C4 and CFB correlated with the local vaginal microbiota. These findings imply that the complement system plays a role in maintaining the proper composition of the vaginal microbiome for a successful pregnancy. The alternative pathway is also important, but not absolute, seeing as the KO mice exhibited normal fertility.

Generally, the CFB is primarily produced by hepatocytes and secreted into the blood, but it is also produced by other type of cells [29]. In the kidneys, the alternative pathway plays an important role in the pathogenesis of lupus nephritis, and the CFB may decrease specific sodium transporter expression during sepsis [30,31,32]. Recently, it was reported that CFB is involved in hypertension and has anticancer activity, due to inhibiting cell proliferation, migration, and age-related macular degeneration [33,34,35]. In addition, KO mice exhibited progressive hearing impairments and reduced choroidal neovascularization development [36,37]. However, it is considered that the deficiency of CFB had no effect on survivability, because there are no reports on lethal phenotypes. We hypothesized that CFB functions in KO mice may be compensated for by other serine proteases. In Figure 4, we demonstrated protease activities in the serum of KO mice. Interestingly, the activities of coagulation factor VIIa and plasmin, measured using Boc-LTR-MCA and Boc-VLK-MCA substrates, was reduced in the serum of KO mice. The coagulation pathway is a proteolytic cascade in hemostasis, initiated by the activated coagulation factor VIIa [38]. This serine protease converts factor X to Xa and factor IX to IXa, which in turn activates further downstream factors that form blood clots. Plasmin is a serine protease that plays a role in the dissolution of blood clots. It is well known that coagulation and complement systems have complicated interactions, and coagulation factor Xa, FXIa, and plasmin can directly cleave C3 and C5 in vitro [39]. Plasmin can initiate C3 activation independently of the classical and alternative pathways [40]. Additionally, plasmin and plasma kallikrein, which are activated by coagulation factor XIIa, cleave CFB into CFBa and CFBb [41]. This suggests that the loss of CFB affects the activity of other serine proteases in the serum as well as homeostatic mechanisms, although we could not identify the protease that could replace CFB.

As shown in Figure 5, the lack of CFB reduced the activity of the classical pathway in the serum, although no significant differences were observed. A previous report showed that serum from KO mice has significantly reduced the activity of classical and alternative pathways [17]. The observation that the removal of CFB also diminishes the activity of the classical pathway is consistent across all these findings. Therefore, it is reasonable to hypothesize that the amplification loop initiated by CFB is implicated not only in the alternative pathway, but also in the classical pathway. In addition, the amplification loop of the alternative pathway is important only when the classical pathway activation is insufficient, due to low antibody titers [42]. These findings suggest that the activation of the alternative pathway is susceptible to changes in the environment and other factors, making it difficult to measure absolute activity, excluding the contributions from other complement pathways. It may be difficult to assess the activity of each complement pathway using in vitro assays with only erythrocytes. Further successful use of complement-deficient mice may reveal biological phenomena, in which the alternative complement pathway is predominant.

## 4. Materials and Methods

### 4.1. Animals

All mice were bred and maintained in cages under a 14 h light and 10 h darkness cycle (room temperature, 24 ± 1 °C; humidity, 50 ± 10%). All animal experiments were performed in an ethical manner after getting approved by the Animal Care Committee of Meiji University (MUIACUC2020-04 and MUIACUC2024-08).

### 4.2. qPCR

The total RNA was extracted using NucleoSpin RNA (MACHE-NAGEL, Dueren, Germany), according to the manufacturer’s instructions. The cDNA was synthesized using SuperScript IV RT (Thermo Fisher Scientific, Waltham, MA, USA). qPCR was performed using the SYBR Green qPCR Master Mix (Thermo Fisher Scientific). The relative mRNA expression in different samples was calculated using the ΔΔCT normalization method. The following primers were used: *Cfb* forward, 5′-GGCAAGCCAAGATCTCAGTC-3′ and reverse 5′-GATCCCTTCTGCCTTTTTCC-3′. *Gapdh* was chosen as an internal control using the following primers: *Gapdh* forward, 5′-TGGCCTTCCGTGTTCCTAC-3′ and reverse 5′-GAGTTGCTGTTGAAGTCGCA-3′.

### 4.3. Immunoblotting

The collected tissues were homogenized in a protein lysis buffer (20 mM Tris/HCl, pH 7.5, 1% Triton X-100, 150 mM NaCl, and 1% protease inhibitor cocktail). The protein concentration was determined using the Bradford method, and all tissue samples were prepared at 1 mg/mL, separated by SDS-PAGE, and transferred onto Immobilon-P membranes (Merck Millipore, Billerica, MA, USA). After blocking with 3% skim milk, the blots were incubated with anti-Factor B Goat pAb (1:2000, Sigma-Aldrich, St. Louis, MO, USA, #341272) overnight at 4 °C, and then with anti-goat IgG antibody conjugated to horseradish peroxidase (1:3000, Sigma-Aldrich). The detection was performed using SuperSignal West Dura Extended Duration Substrate (Thermo Fisher Scientific). The signal intensities were quantified using ImageJ software version 1.54d.

### 4.4. Preparation of Reagent Used for i-GONAD

Two gRNAs targeting the *Cfb* were prepared using the UCSC Genome Browser (https://genome.ucsc.edu/index.html, accessed on 1 June 2023) and CHOPCHOP (https://chopchop.cbu.uib.no, accessed on 1 June 2023). The sequence of crRNA1 is 5′-TGGAGTTGCGCTCACACCTGAGG-3′ at exon 2, and that of crRNA2 is 5′-AGACACCACGGCCCCCATACAGG-3′ at exon 12. crRNAs and tracrRNA were purchased from Integrated DNA Technologies, Inc. (IDT; Coralville, IA, USA), and prepared for 3 μg/μL for annealing to allow the formation of crRNA/tracrRNA duplexes (gRNA). gRNA was mixed with 1 μL of 10 μg/μL Alt-R S.p. Cas9 Nuclease 3NLS (IDT), 1 μL of FASTGREEN (nacalai tesque, Inc., Kyoto, Japan), and 7 μL of Opti-MEM (Thermo Fisher Scientific) and stored at −80 °C until use.

### 4.5. i-GONAD

*i*-GONAD was performed as previously described [18]. The estrous cycle of the C57BL/6N mice was determined by the appearance of vaginal smear, and the animals were mated the day before surgery. The following morning, the mice were checked for vaginal plugs, and *i*-GONAD was performed at 4 pm on the same day. The gRNA solution was injected into the oviduct lumen upstream of the ampulla, followed by electroporation using a CUY21EDITII (BEX, Tokyo, Japan) instrument.

### 4.6. Genotyping and Sequencing

The genotyping was performed by PCR, using KOD-FX neo (TOYOBO, Osaka, Japan), and the primer pair flanked the knockout region. The sequence of the primer F is 5′-ATCCAGCATTTGGGTTTCAG-3′ at exon 1, and that of primer R is 5′- CCAGGCCTTGAACTAAGCAG-3′ at intron between exon 14 and 15, the expected size of PCR products was 4815 bp from WT allele and 977 bp from KO allele (Figure 1c). PCR products on gels were cloned and sequenced using the Big Dye Terminator v3.1 Cycle Sequencing Kit (Thermo Fisher Scientific).

### 4.7. Mouse Fertility In Vivo and In Vitro

To evaluate the KO mice’s fertility in vivo, the number of pups delivered from 8- to 12-week-old female mice was recorded after a 1 week mating period, during which female mice were housed with 8- to 12-week-old male mice.

Sperm concentration and motility parameters in vitro were quantified using a sperm motility analysis system (DITECT, Tokyo, Japan). Cauda epididymal sperm were dispersed for 10 min and incubated for 1, 2, and 3 h in a TYH-medium, at 37 °C, under 5% CO_2_ in air. After the incubation, 2 μL aliquots of the sperm suspension was transferred to a sperm chamber, and more than 200 sperm were examined for each sample.

The spontaneous sperm acrosome reaction in vitro was evaluated by staining with PNA-FITC (Sigma-Aldrich), as described previously [43]. After incubation in vitro, sperm were air-dried, permeabilized, and fixed in 100% methanol for 10 min at room temperature. After washing with PBS, the sperm were incubated with 100 µg/mL PNA-FITC in PBS for 30 min, at 37 °C, and washed three times with PBS.

The number of ovulated oocytes was counted to evaluate the females’ fertility. Oocyte-cumulus complexes, containing the metaphase II-arrested oocytes, were collected from the oviductal ampulla of the superovulated mice, 14 h after the hCG injection. After the incubation with hyaluronidase, denuded eggs were placed in TYH medium covered with liquid paraffin.

### 4.8. Analysis of Gut and Vaginal Microbiota

Vaginal lavages were collected from the female mice at the estrus stage by pipetting 10 μL of sterilized PBS. Stool samples were collected from the same mice that the vaginal samples were taken from, and the total DNA of the stool microbiota was extracted using the QIAmp Fast DNA Stool Mini Kit (QIAGEN, Hilden, Germany). All samples were subjected to 16S rRNA gene amplicon sequencing analysis at Genome-Lead Co., Ltd. (Takamatsu, Kagawa, Japan), using the Illumina MiSeq platform and MiSeq Reagent Micro Kit v2 (Illumina, San Diego, CA, USA). Raw data were visualized and analyzed using QIIME2 (https://qiime2.org, accessed on 15 December 2024).

### 4.9. Enzyme Assay

The enzymatic activity was measured using 4-methylcoumaryl-7-amide (MCA) substrate (Peptide Institute Inc., Osaka, Japan) as described previously [44]. The reaction mixture (125 μL) consisted of 100 mM Tris/HCl (pH 7.5), 150 mM NaCl, and 40 μM substrate. Reactions were performed at 37 °C, for 1 h, and the released 7-amino-4-methyl coumarin was measured every 10 min for 1 h, with the excitation at 380 nm and the emission at 460 nm, using SpectraMax iD5 (Molecular Devices, San Jose, CA, USA). The enzymatic activity in relative fluorescence units per second (RFU/s) was calculated from the initial rate of the reaction.

### 4.10. Hemolytic Assay

To measure the hemolytic activity of the classical complement pathway, sheep blood in Alsevers (ROCKLAND, Philadelphia, PA, USA) was prepared for the assay by gentle washing in HEPES Buffered Saline (HBS; containing 10 mM HEPES, pH7.6, 150 mM NaCl, 135 μM CaCl_2_, 1 mM MgCl_2_), followed by sensitization with rabbit anti-sheep erythrocyte (Nordic-MUbio, Susteren, The Netherlands) at 37 °C, for 30 min. The sensitized ShE (ShEA) were washed with HBS and incubated with 16.7% mouse serum at 37 °C for 30 min in a tube. ShEA was incubated with deionized water as the positive control (100% lysis) or HBS as the negative control (0% lysis). Following incubation, reaction tubes were centrifuged at 1500× *g* at 4 °C for 3 min, and the supernatants were transferred to a Microplate 96-well (WATSON, Tokyo, Japan). Absorbance was measured at 405 nm using SpectraMax iD5. The percentage of hemolysis was calculated as follows: (Sample A405 − negative control A405)/(positive control A405 − negative control A405) × 100.

To measure the hemolytic activity of the alternative complement pathway, sterile whole rabbit blood (Nippon Bio-Supp. Center, Tokyo, Japan) was gently washed with HBS-MgEGTA (10 mM HEPES, pH7.6, 150 mM NaCl, 135 μM CaCl_2_, 3 mM MgCl_2_, 5 mM EGTA). Mouse serum, diluted to 25% with HBS-MgEGTA, was subjected to the hemolysis assay without an IgG sensitization.

### 4.11. Statistical Analysis

All statistical analyses were performed using the GraphPad Prism software (GraphPad Software Inc., San Diego, CA, USA). Experiments were independently performed at least three times under similar conditions. All statistical results were presented as the mean ± standard error of the mean and calculated using the two-tailed, unpaired Student’s *t*-test in GraphPad Prism version 9.0. P-value < 0.05 was considered statistically significant.

## Data Availability

The original contributions presented in this study are included in the article/Supplementary Materials. Further inquiries can be directed to the corresponding author.

## Supplementary Materials

The supporting information can be downloaded at: https://www.mdpi.com/article/10.3390/ijms26031393/s1.
