# Supplementary figures and images for: Complement Factor B Deficiency Is Dispensable for Female Fertility but Affects Microbiome Diversity and Complement Activity
Source: Int J Mol Sci. 2025 Feb 6;26(3):1393. doi: 10.3390/ijms26031393 (PMC11818189; doi:10.3390/ijms26031393)

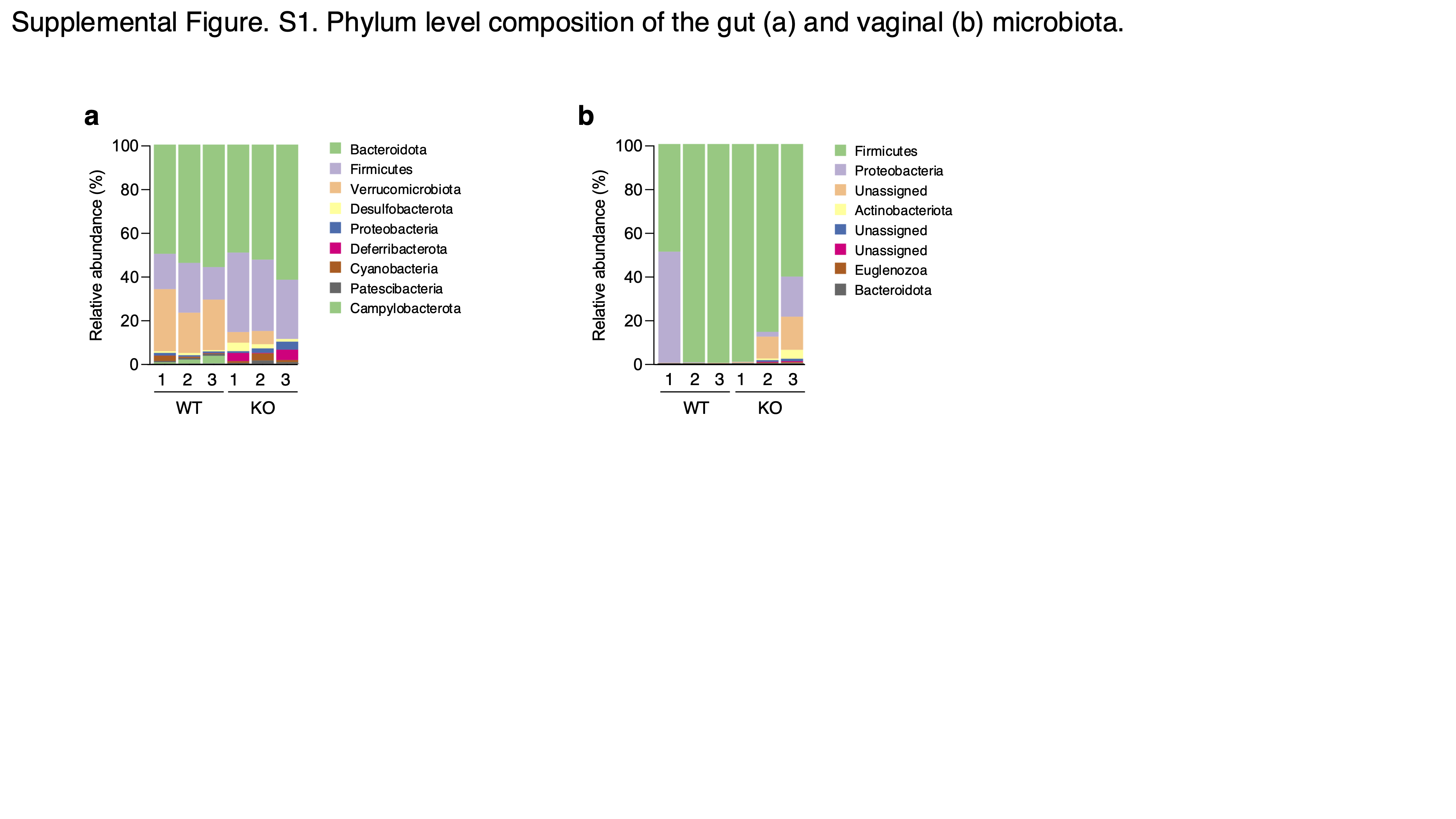

Supplement: Supplementary file 1 [file ijms-26-01393-s001.zip › Supplemental Figure S1.tiff]

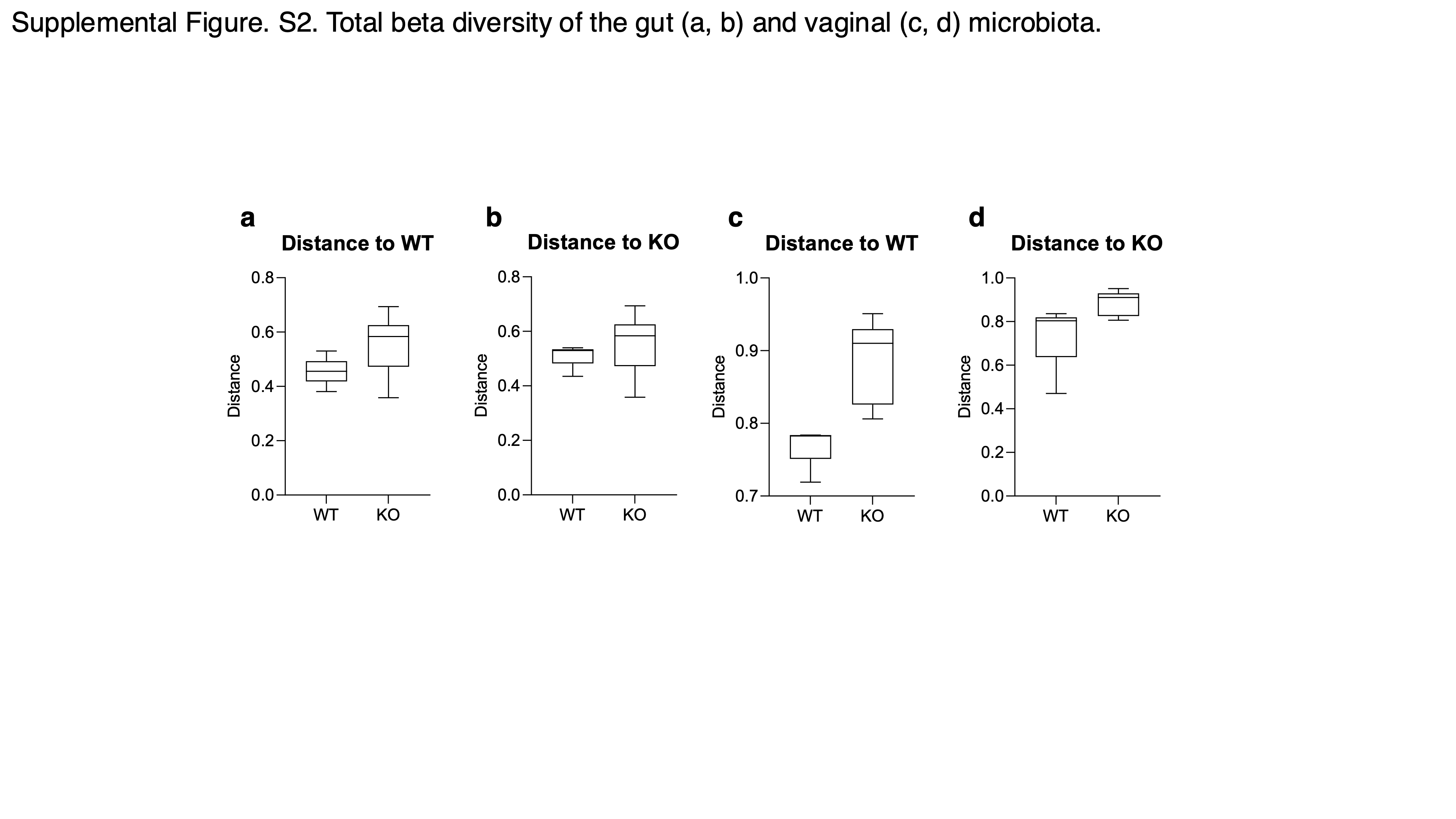

Supplement: Supplementary file 1 [file ijms-26-01393-s001.zip › Supplemental Figure S2.tiff]

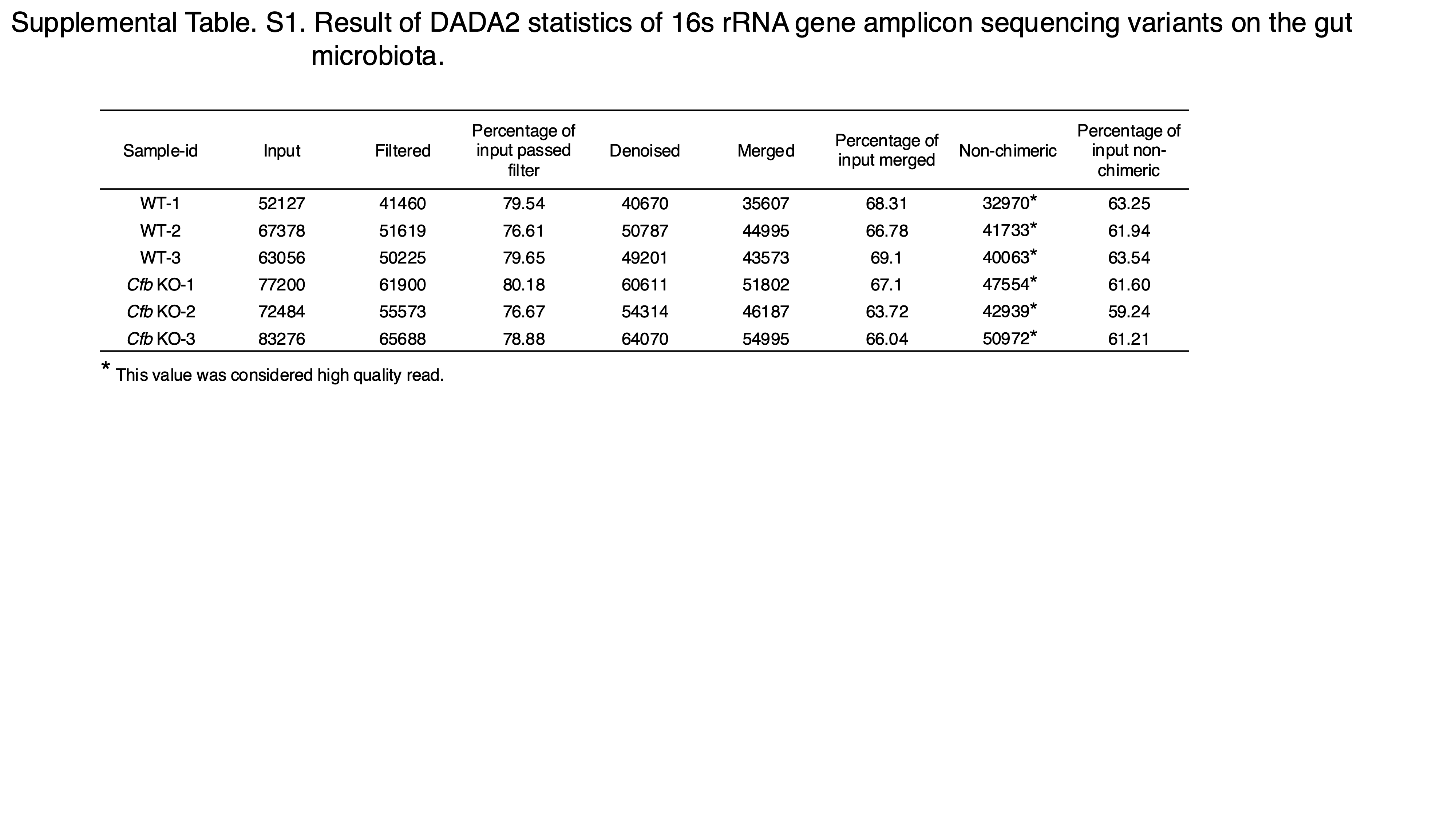

Supplement: Supplementary file 1 [file ijms-26-01393-s001.zip › Supplemental Table S1.tiff]

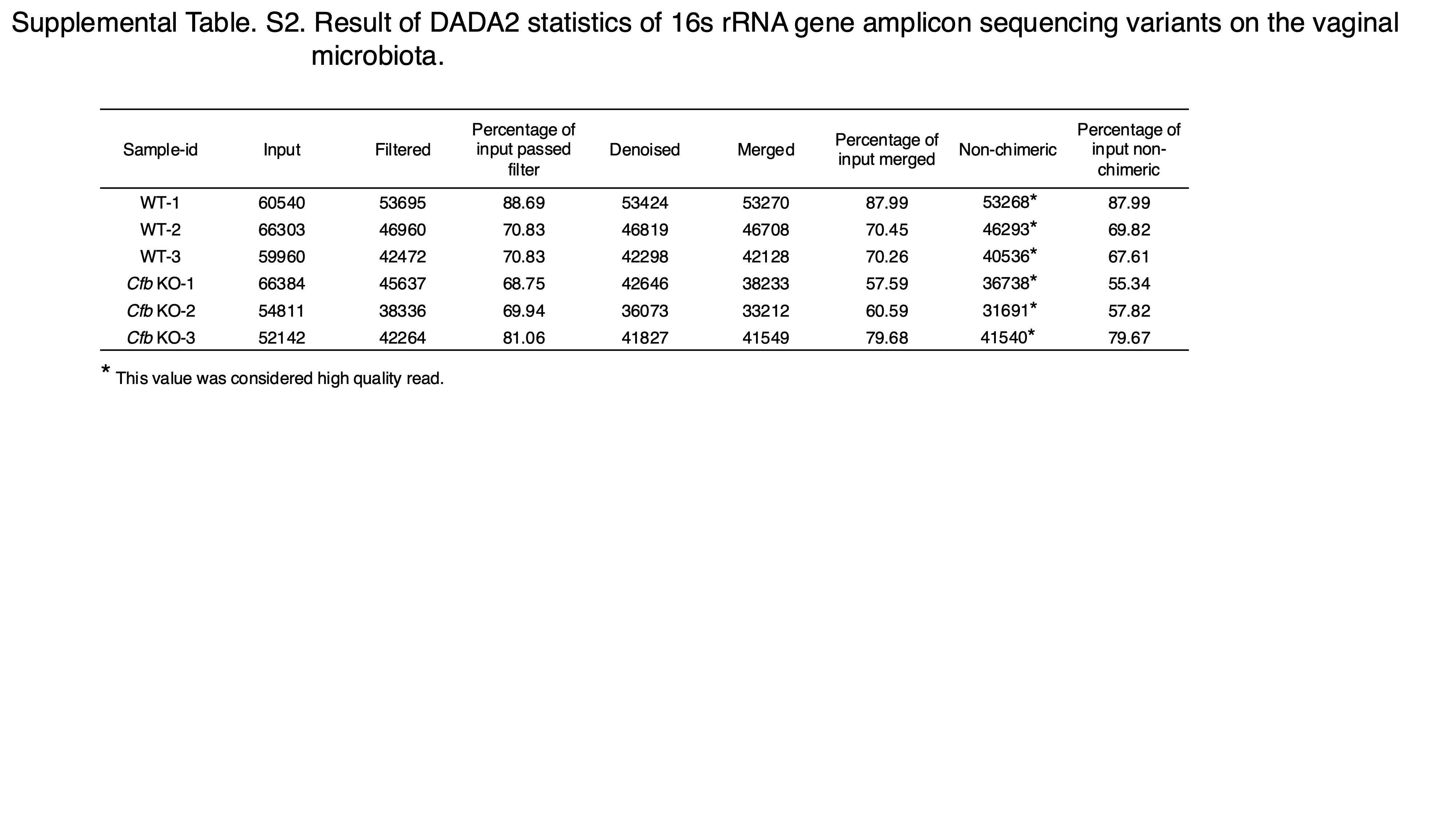

Supplement: Supplementary file 1 [file ijms-26-01393-s001.zip › Supplemental Table S2.tiff]

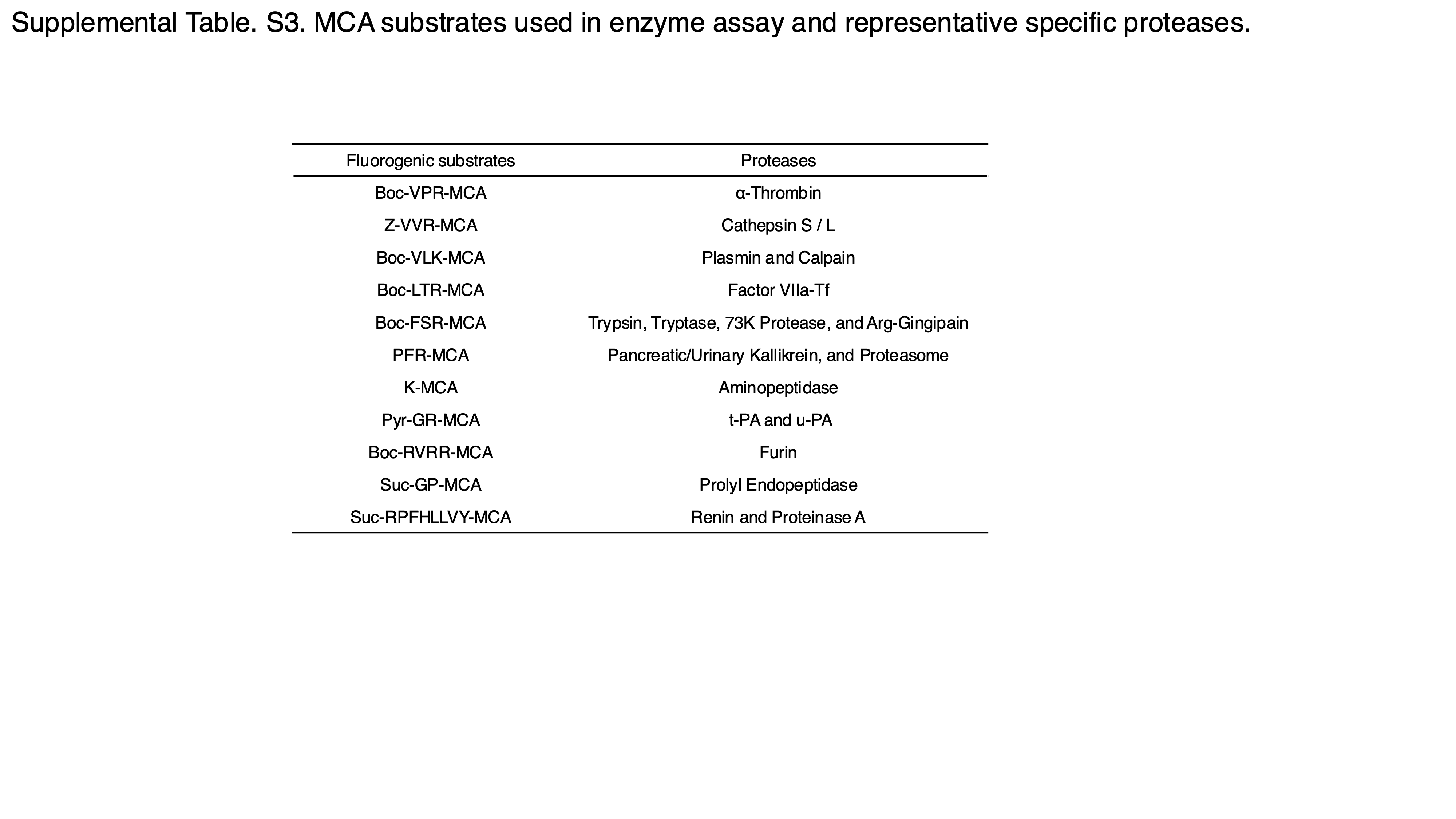

Supplement: Supplementary file 1 [file ijms-26-01393-s001.zip › Supplemental Table S3.tiff]
